# Alterations in the hepatocyte epigenetic landscape in steatosis

**DOI:** 10.1186/s13072-023-00504-8

**Published:** 2023-07-06

**Authors:** Ranjan Kumar Maji, Beate Czepukojc, Michael Scherer, Sascha Tierling, Cristina Cadenas, Kathrin Gianmoena, Nina Gasparoni, Karl Nordström, Gilles Gasparoni, Stephan Laggai, Xinyi Yang, Anupam Sinha, Peter Ebert, Maren Falk-Paulsen, Sarah Kinkley, Jessica Hoppstädter, Ho-Ryun Chung, Philip Rosenstiel, Jan G. Hengstler, Jörn Walter, Marcel H. Schulz, Sonja M. Kessler, Alexandra K. Kiemer

**Affiliations:** 1 Institute for Cardiovascular Regeneration, Goethe-University, 60590 Frankfurt, Germany; 2 German Centre for Cardiovascular Research (DZHK), Partner Site Rhine-Main, 60590 Frankfurt, Germany; 3 Department of Pharmacy, Pharmaceutical Biology, Saarland University, 66123 Saarbrücken, Germany; 4 Centre for Genomic Regulation (CRG), Barcelona Institute of Science and Technology (BIST), 08003 Barcelona, Spain; 5 Department of Genetics, Saarland University, 66123 Saarbrücken, Germany; 6 IfADo: Leibniz Research Centre for Working Environment and Human Factors, Dortmund, Germany; 7 Department of Computational Molecular Biology, Max Planck Institute for Molecular Genetics, 14195 Berlin, Germany; 8 Institute of Medical Bioinformatics and Biostatistics, Philipps University of Marburg, 35032 Marburg, Germany; 9 Institute of Clinical Molecular Biology, Christian-Albrechts-University, 24105 Kiel, Germany; 10 Core Unit Bioinformatics, Medical Faculty, Heinrich Heine University, 40225 Düsseldorf, Germany; 11 Department of Computational Biology and Applied Algorithmics, Max Planck Institute for Informatics, 66123 Saarbrücken, Germany; 12 Excellence Cluster on Multimodal Computing and Interaction, Saarland University, 66123 Saarbrücken, Germany; 13 Institute of Pharmacy, Experimental Pharmacology for Natural Sciences, Martin Luther University Halle-Wittenberg, Halle, Germany; 14 Halle Research Centre for Drug Therapy (HRCDT), Halle, Germany

## Abstract

Fatty liver disease or the accumulation of fat in the liver, has been reported to affect the global population. This comes with an increased risk for the development of fibrosis, cirrhosis, and hepatocellular carcinoma. Yet, little is known about the effects of a diet containing high fat and alcohol towards epigenetic aging, with respect to changes in transcriptional and epigenomic profiles. In this study, we took up a multi-omics approach and integrated gene expression, methylation signals, and chromatin signals to study the epigenomic effects of a high-fat and alcohol-containing diet on mouse hepatocytes. We identified four relevant gene network clusters that were associated with relevant pathways that promote steatosis. Using a machine learning approach, we predict specific transcription factors that might be responsible to modulate the functionally relevant clusters. Finally, we discover four additional CpG loci and validate aging-related differential CpG methylation. Differential CpG methylation linked to aging showed minimal overlap with altered methylation in steatosis.

**Supplementary Information:**

The online version contains supplementary material available at 10.1186/s13072-023-00504-8.

## Introduction

Epigenetic changes have gained attention in recent research focusing on liver diseases [1]. Metabolic dysfunction-associated fatty liver disease (MAFLD) affects about a quarter of the global population and comprises a spectrum of liver pathologies that arise from different etiologies, all being characterized by hepatic steatosis. MAFLD increases the risk for the development of fibrosis, cirrhosis, and hepatocellular carcinoma. Fatty liver disease has traditionally been subdivided into the terms alcoholic fatty liver disease (AFLD) or non-alcoholic fatty liver disease (NAFLD) based on rather arbitrary cut-off amounts of patients’ daily alcohol consumption. The overarching term MAFLD has therefore been suggested in order “to integrate the current understanding of patient heterogeneity” [2]. Interestingly, the majority of genes dysregulated in livers of alcoholic steatohepatitis (ASH) and non-alcoholic steatohepatitis (NASH) patients has been reported to be identical [3].

The epigenome modulates gene expression changes through different mechanisms, such as histone modifications, DNA methylation, and non-coding RNA-mediated actions. Post-translational modifications of histones affect gene transcription by altering the DNA accessibility to the transcriptional machinery. These aberrant histone modifications have been shown to be associated with the development of insulin resistance and consequently NAFLD [4]. Exposure to ethanol has also been found to cause an imbalance of histone acetylation and deacetylation enzymes in hepatocytes. H3K9 acetylation was found to correlate with a transcriptional increase of alcohol dehydrogenase (ADH1) [5]. Furthermore, ethanol was reported to be a stimulator of fibrosis by altering histone-modifying enzymes in hepatic stellate cells (HSCs), resulting in increased expression of extracellular matrix proteins including elastin [6, 7]. Ethanol also has been reported to induce global DNA hypomethylation and an aberrant pattern of DNA methylation [7]. Knowledge on ethanol-induced epigenetic alterations have typically been generated either by in vitro treatment of cell cultures or by analyses of whole tissue. We are not aware, until this study was conducted, of any epigenetic studies employing ex vivo material, i.e., pure cell preparations from animals undergoing a feeding scheme [7]. There is also a lack of knowledge of epigenetic alterations in isolated hepatocytes for NAFLD.

DNA methylation (DNAm) is predominantly associated with chromatin condensation, inhibiting the binding of transcriptional activators, and thereby resulting in transcriptional silencing [8, 9]. CpG islands are preferentially found in an unmethylated state in promoters and are associated with transcriptionally active states. Different methylation patterns have been proposed to distinguish different stages of NAFLD or fibrosis [10]. Comparing mild and advanced NAFLD in liver biopsies of patients, a general trend in hypomethylation of CpG sites was observed in advanced NAFLD [11]. Loomba et al. [12] described interesting DNAm signatures in the peripheral blood cells of nonalcoholic steatohepatitis (NASH) patients showing epigenetic age acceleration. Horvath et al. [13] found an increased epigenetic age in liver tissues from obese individuals and indications that body mass index might promote age acceleration via steatosis.

In this study, we aimed to (i) elucidate the features caused by epigenetic alterations in hepatocytes isolated from steatotic animals fed the Lieber DeCarli (LDC) diet (containing high fat and alcohol) by an integrative approach of analyzing full class epigenomes. (ii) assess whether methylation marks of the epigenetic clock, as suggested by Stubbs et al. for murine tissues [14], could be observed in these isolated cells. Through this work, we find four functionally relevant clusters of genes differentially expressed in the LDC model, which are regulated by chromatin changes and transcription factors. This study reveals that LDC-induced steatosis does not correlate with DNAm age.

## Materials and methods

### Animal welfare

Animal handling was in compliance with the guidelines of the local animal welfare committee (permission number: 38/2013). Details on mouse treatment were described in [15]. Mice were housed in a 12/12 h light/dark cycle under constant conditions (temperature: 22 °C ± 2 °C; relative humidity: 55% ± 10%) with food and water ad libitum. Female control C57Bl6/JxDBA/2 were randomly divided into the experimental groups at the age of 3 weeks. The control group (Co) received normal chow (#1320, Altromin, Lage, Germany). The other group was fed the Lieber-DeCarli (LDC, #F1258SP, BioServ, Flemington, NJ, USA) diet as the only food source. The composition of the diets with regard to metabolic energy was 24% kcal from protein, 12% kcal from fat, and 64% from carbohydrates for the Co diet and 17.2% kcal from protein, 40.9% kcal from fat, 15.4% from carbohydrates, and 26.5% from ethanol, which equals 4% ethanol, for the LDC diet. Mice were sacrificed at the age of 9 weeks.

The diet was prepared as recommended by the manufacturer’s instructions and animals were fed as published [15, 16] with a magnetic stirrer and a magnetic stir bar. To one-third of the dry mix one-third of warm water was added, and mixed until the product dispersed. This step was repeated, ethanol was added and the product was dispensed into liquid diet feeding tubes (#13260, BioServ, Flemington, NJ, USA). Mice received the LDC diet for 1 week without ethanol, followed by 1 week of increasing ethanol concentrations: 2 days 1%, 2 days 2%, 3 days 4%. The lipid composition induced by LDC feeding was analyzed in mice treated in parallel to the animals used for hepatocyte isolation within this project and confirmed massive lipid deposition [15]. For analysis of DNA methylation related to aging, hepatocytes were isolated from female control C57Bl6/J mice at the age of 10 weeks (young) and 40 weeks (mid-aged); for DNA methylation analyses in liver tissues, livers from female control mice aged 10 (young) and 84–85 (aged) weeks were used [17].

#### Hepatocyte isolation

Isolation of primary hepatocytes from mouse livers was performed as described previously [18]. In brief, the liver was perfused through the vena cava with an EGTA-containing buffer, followed by a perfusion with collagenase buffer. After digestion, the liver was excised and the liver capsule was opened under sterile conditions, and the cells were released into a suspension buffer. The cell suspension was filtered through a 100 µm gauze to remove tissue debris and centrifuged for 5 min at 4 °C and 50×*g*. The hepatocyte pellet was washed and the centrifugation step was repeated. Aliquots of hepatocytes were cryopreserved and stored at − 80 °C until further analysis.

### Colorimetric Sulfo-Phospho-Vanillin assay

The colorimetric Sulfo-Phospho-Vanillin assay was used to quantify total lipids in isolated hepatocyte samples. Freeze-dried samples were dispersed with 18 volumes of hexane/2-propanol (3:2 (v/v)) for 10 min and centrifuged for 10 min at 4 °C and 10,000×*g*. The supernatant was transferred into a new glass vial (#60500-1109, DURATEC Analysentechnik GmbH, Hockenheim, Germany), dried under nitrogen stream, re-dissolved in 200 µl chloroform–methanol (2:1 (v/v)), and stored at − 20 °C. As a standard solution olive oil was diluted in chloroform–methanol (2:1 (v/v)). 100 µg, 75 µg, 50 µg, 25 µg, 12.5 µg and 6.25 µg olive oil were used as a standard and handled like the samples. 5 µl of the lipid extracts were transferred into a 1.5 ml glass vial and the solvent was evaporated by incubation for 2 to 5 min at 90 °C in a drying closet. Samples were cooled to room temperature, 100 µl of sulfuric acid (95–97%, #100731.1000, Merck, Darmstadt, Germany) was added and incubated for 20 min at 90 °C. After cooling the vials down to room temperature, 50 µl vanillin-phosphoric acid (0.2 mg vanillin per ml 17% orthophosphoric acid (85%, #20624, VWR, Darmstadt, Germany) was added, followed by 10 min incubation at room temperature. 100 µl of the colored solution was transferred to a 96 well plate and the absorption was measured at 550 mm using the Sunrise™ absorbance microplate reader (Tecan Austria GmbH, Grödig, Austria).

### RNA sequencing

RNA was isolated using the TRIzol method. Briefly, 1 ml TRIzol was added to 5,000,000 cells followed by vortexing, a 5-min incubation at room temperature, and addition of 200 μl chloroform. After mixing, further incubation at room temperature for 2–3 min and centrifugation (12,000*g*) at 4 °C for 5 min, the clear supernatant was mixed with 500 μl isopropanol and incubated at room temperature for 10 min. After further centrifugation (12,000*g*) at 4 °C for 10 min, the supernatant was discarded and the pellet washed with 1 ml cold 75% ethanol followed by vortexing and centrifugation (7500*g*, 4 °C, 5 min). The pellet was dried and dissolved in RNase-free water. The quality (QC) of total RNA as determined by TapeStation or Bioanalyzer (Agilent) was above RIN 9.5. For the preparation of the libraries, rRNA was removed with the Ribo-Zero™ Human/Mouse/Rat rRNA Removal Kit from Biozym Scientific GmbH.

The long RNA library was prepared using the TruSeq Stranded totalRNA Sample Prep Kit from Illumina (San Diego, CA) according to the manufacturer’s instructions. All samples were sequenced using an Illumina HiSeq 2500 sequencer v4 (Illumina, San Diego,CA) (1 sample/lane plus 2 × 125 bp for long RNA) at IKMB NGS core facilities.

### Capturing DNA methylation

#### Bisulfite treatment and PCR

500 ng genomic DNA was subjected to bisulfite treatment using the EZ DNA Methylation-Gold Kit (Zymo Res.) according to the manufacturer's protocol. Two microliters of bisulfite treated DNA was used as template in a 30-μL reaction in the presence of 3 mM of Tris–HCl (pH 8.8), 0.7 mM of (NH_4_)_2_SO_4_, 50 mM of KCl, 2.5 mM of MgCl_2_, 0.06 mM of each dNTP, 3 U HotFire DNA polymerase (Solis BioDyne), and 167 nM of primers (Supp. Table 1). PCRs were performed at 95 °C for 15 min followed by 42 cycles at 95 °C/60 s, 54 °C (58 °C for Tns1 and Art3 reactions)/30 s, 72 °C/30 s, and a final extension 72 °C/5 min.

#### SNuPE/HPLC analysis

Primer extension was performed as previously described [19]. Five μl of PCR products were treated with 1U of ExoCIAP (mixture of Exonuclease I [Jena Bioscience] and Calf Intestine Alkaline Phosphatase [Calbiochem]) for 30 min at 37 °C. To inactivate the ExoCIAP enzymes, the reaction was incubated for 15 min at 80 °C. Afterwards, 14 μl of primer extension mastermix (50 mM of Tris–HCl, pH 9.5, 2.5 mM of MgCl_2_, 0.05 mM of ddNTPs, 1.6 μM of each SNuPE primer (Additional file 1: Table S1), and 2.5 U of Termipol DNA polymerase [Solis BioDyne]) were added. Primer extension reactions were performed at 96 °C for 2 min, followed by 50 cycles at 96 °C/30 s, 50 °C/30 s, and 60 °C/20 s. Separation of products was conducted on an XBridge BEH C18 2.5 µm 4.6 mm × 50 mm column (Waters) at 0.9 ml/ min at 50 °C by continuously mixing buffer B (0.1 M TEAA, 25% acetonitril) with buffer A (0.1 M TEAA) (Additional file 1: Table S1). Methylation indices were determined by measuring the height (h) of the methylated (M) and unmethylated (UM) peak using the equation h(M)/(h(M) + h(UM)).

#### Reduced representation bisulfite sequencing (RRBS)

Library preparation was conducted as described previously in a one-tube reaction [20]. Briefly, 500 ng genomic DNA was digested with *Msp*I, repaired and A-tailed using Klenow fragment enzyme (NEB), subsequently Illumina TruSeq universal adaptors were ligated (T4 ligase, NEB). Bisulfite treatment was performed using the EZ DNA Methylation-Gold Kit (Zymo Res.) according to the manufacturer's protocol. Library preparation was accomplished by amplification with indexed TruSeq adaptor sequences (12 cycles) to add sample-specific 6 bp identifiers. Sequencing was conducted on the HiSeq2500 (Illumina) on a 100 bp single-read flow cell aiming at 25–30 Mio reads per sample.

### DNaseI sequencing

DNase I sequencing (DNase-seq) was performed as previously described [21, 22]. Briefly, nuclei were isolated from 1 × 10^7 cells by using buffer A (60 mM KCl, 15 mM Tris–HCl (pH 8.0), 15 mM NaCl, 1 mM EDTA (pH 8.0), 0.5 mM EGTA (pH 8.0), 0.5 mM spermidine free base) supplemented with IGEPAL (0.1% final concentration) and incubation on ice for 15 min. Nuclei were treated with different DNase I concentrations (2,25 × 10^6 nuclei each, with 0–80U/ml) for 3 min at 37 °C and the reaction was stopped at 55 °C for 1 h with stop buffer (50 mM Tris–Cl (pH 8.0), 100 mM NaCl, 0.1% SDS, 100 mM EDTA (pH 8.0), 1 mM spermidine and 0.3 mM spermine) supplemented with proteinase K (50 µg/ml). DNA was then purified using phenol chloroform extraction and double-hit fragments of 100–500 bp were selected by sequential purifications with Agencourt AMPure XP Beads (Beckman Coulter, Brea, USA). Sequencing libraries were prepared from 8 ng of purified DNA (from 80 U/ml-digest) using the TruSeq ChIP Library Preparation kit (Illumina, San Diego, USA) according to the manufacturer’s protocol and sequenced on an Illumina HiSeq2500 (v3 paired-end flow cell). Raw reads were processed with the DEEP pipelines GALv1 and DHSv3 [23] (https://github.molgen.mpg.de/DEEP/comp-metadata).

### Chromatin Immunoprecipitation (ChIP sequencing)

Cells were crosslinked in 1% formaldehyde for 5 min at room temperature under rotation, followed by quenching in 0.125 M glycine for 10 min. The crosslinked cells were then pelleted by centrifugation for 5 min at 4 °C. ChIP seq was performed according to the Nexson protocol [24]. In brief 625,000 cells were used per ChIP. Cell were first lysed in 500 μl of Farmen Buffer (5 mM PIPES pH8, 85 mM KCl, 0.5% Igpal, 1 × complete protease inhibitor) and then briefly sheared on a Diagenode Bioruptor Plus for 6 cycles (15 s ON/30 s OFF), to free nuclei. Nuclei were isolated by brief centrifugation at 2000 rpm for 5 min at 4 °C. Nuclei were then lysed and resuspended by homoginization with a 27 guage syringe in 440 μl of 0.33% SDS shearing buffer (100 mM NaCl, 50 mM Tris–HCl pH8.1, 0.2% NaN3, 0.33%SDS, 3% Triton X-100) and divided into 4 tubes for shearing on a Bioruptor Pico for 45 cycles (30 s ON/ 30 s OFF) on high frequency. The chromatin was then diluted to 0.11% SDS prior to peforming the ChIP on the IPstar with (50 mM Tris–HCl pH8.6, 100 mM NaCl, 5 mM EDTA pH 8.0, 0.2% NaN3). ChIPs were performed on the Diagenode IPstar automated machine using the following set up parameters: Indirect method, the Auto Histone ChIP Kit (Diagenode)-200 μl, 1 μg antibody—Diagenode H3K27ac (pAb-196–050), H3K27me3 (pAb-195–050), H3K36me3 (pAb-192–0500), H3K4me1 (pAb-194–050), H3K4me3 (pAb-003–050), H3K9me3 (pAb-193–050) and 10 h antibody incubation, 5 h bead incubation, 5 min washes. The ChIPs were then de-crosslinked on the IPstar for 4 h at 65∙C. ChIP samples were then removed and treated with 2 µl RNase A (10 mg/ml) 30 min at 37 °C followed by 3ul Proteinase K treatment for 3 h at 55 °C. DNA was purified with Zymo concentrator ChIP DNA clean up columns. ChIP DNA was then quantified by Quibit. ChIP libraries were generated using NEBNext® Ultra DNA Library Prep Kit for Illumina® (E7370S/L) according to the manufacturer’s instructions. The PCR cycles were as follows: 5 Steps: 1 Cycle: 98 °C for 30 s; 10 Cycles: 98 °C for 2 min; 1 Cycle: 98 °C for 10 s and 65 °C for 75 s; 1 Cycle: 65 °C for 5 min; Hold at 4 °C. The libraries were paired-end sequenced on an Illumina HiSeq 2500 platform.

### Bioinformatic analyses

#### Differential gene expression analysis

RNA reads were trimmed for adapter and low-quality tails (Q < 20) with TrimGalore (http://www.bioinformatics.babraham.ac.uk/projects/trim_galore/) and subsequently aligned to the mm10 reference genome with gene models from GENCODE (version M2) 11 by the IHEC supported pipeline grape-nf (https://github.com/guigolab/grapenf/tree/35e44730f5da02a41e2aef7d97a722e20c5773f). tGrape-nf wraps STAR (version 2.4.0j)12 and RSEM (version 1.2.21)13.

DESeq2 (Version 1.18.1) was used to detect differentially expressed genes (DEGs) with maximal adjusted *p*-value of 0.05. Genes for which less than four samples had an FPM (fragments per million mapped reads) above 0.1 were discarded.

Differentially expressed genes (DEGs) with adjusted p < 0.05 were used for GO functional (MF, BP and CC) and biological pathway enrichment (KEGG, Reactome and WikiPathways) using g:Profiler [25].

Moreover, these DEGs were used to build the PPI network using STRING v11 [26]. The active interaction sources were taken from literature, experiments, databases, co-expression and co-occurrences of the nodes (or proteins). A confidence score (> 0.7) was chosen to get the interacting nodes. Following this, k-means clustering of the genes was performed. The optimal number of clusters (k = 4) was chosen based on the significant association and enrichment of GO terms and KEGG pathways for the genes (in each cluster).

#### Differential DNase peak analysis

TEPIC v2 [27] was used to compute the TF gene scores based on the differential DNase peaks regions for LDC over Co (pseudocount set to 1 e-6). A base mean cutoff of 10 was used to filter for expressed TFs from the results of differential gene expression analysis. This resulted in 265 motifs, out of 380 total motifs from JASPAR [28], HOCOMOCO [29] and KELLIS *ENCODE Motif database* [30] (included in TEPIC v2). The Differential DNase I peaks were obtained using edgeR [31]. The positive and negative peaks for the steatosis (LDC) vs control (Co) were identified based on positive log fold change i.e. logFC > 0 (and vice versa). GENCODE (vM21) was used for reference genome sequence annotations.

#### Prediction of transcription factors involved in gene regulation

DYNAMITE (from TEPIC v2 [27] package) was then used (parameters: –Ofolds = 10 –Ifolds = 6 –alpha 0.01) to identify the transcriptional regulators that regulate the differentially expressed genes (LDC over Co). The TFs were ranked based on absolute regression coefficient, and the top 15 TFs (absolute coefficient >  = 0.125) were used for further analysis. The TF effect score (or TF enrichment) is calculated (as in Eqs. 1 and 2) by the difference of the median log2 quotient *Q*_*t,i*_ (for each TF, obtained from the TF gene score) for all the DE genes in each cluster and mean of the median Log2 quotients (of others) (Fig. 2b).

1 $${Q_{t,i}} = Median\left\{ {{{\log }_2}\frac{{A_{t,g}^{LDC}}}{{A_{t,g}^{Co}}};\,\,\forall \,\,g \in cluste{r_i}} \right\}$$

2 $${E_{t,i}} = {Q_{t,i}} - \frac{1}{j}\sum\limits_{j,j \ne i}^{} {{Q_{t,j}}}$$

where, *A*_*t,g*_ is the TF affinity score for TF *t* regulating gene *g*. The affinity score of a gene is the sum of TF affinity values for all open-chromatin regions (peaks) in LDC or Co in a window around gene *g*. *E*_*t,i*_ is the TF effect score for transcription factor *t* in cluster *i*.

#### Differential chromatin domain annotation

Chromatin state segmentation tracks were computed using ChromHMM (v1.15) [32] with default parameters using the 18-state model published by the Roadmap Epigenomics Consortium [33] for the ChIP-seq data of the six histone modifications H3K36me3, H3K27ac, H3K27me3, H3K9me3, H3K4me3, H3K4me1 plus the Input control. The input short-read alignment (BAM) files were filtered with *sambamba* (v0.6.8) [34] to retain only properly paired reads that were not marked as duplicate or supplementary alignments, and that were aligned with a mapping quality (MAPQ) of at least 5 as reported by the read aligner.

Next, we used SCIDDO (development version #39de43a) [23] to identify larger domains of chromatin differences among the two conditions control (Co) and steatosis (Ldc). We executed SCIDDO with default parameters, using the emissions of the 18-state ChromHMM model (see above) to set the scoring scheme. The identified differential chromatin domains (DCDs) were then reduced to domains unique to the respective condition of interest (using bedtools v2.27.1 [35], command “intersect -v”), and the unique domains further intersected with gene bodies of protein-coding genes (GENCODE vM21, [36]) to obtain a list of genes putatively affected by differential chromatin marking.

#### Analysis of RRBS data

Raw DNA methylation data for hepatocytes and whole liver has been processed by the pipeline implemented within the DEEP project (https://github.molgen.mpg.de/DEEP/comp-metadata) to generate BED files. These files were used as input to *RnBeads* [37] for further analysis. We filtered for CpGs that are covered by at least 5 sequencing reads and conducted differential analysis between the aged and young samples using the *limma* method [38] as implemented in *RnBeads* [37]. The resulting *p*-values were corrected for multiple testing using the Benjamini–Hochberg method and the following criteria were used to select differentially methylated CpGs: mean methylation difference of at least 0.05, methylation variance in both of the groups less than 0.05 and a FDR-adjusted *p*-value less than 0.05.

## Results

### Generation of epigenome and expression data of hepatocytes

**Fig. 1:**
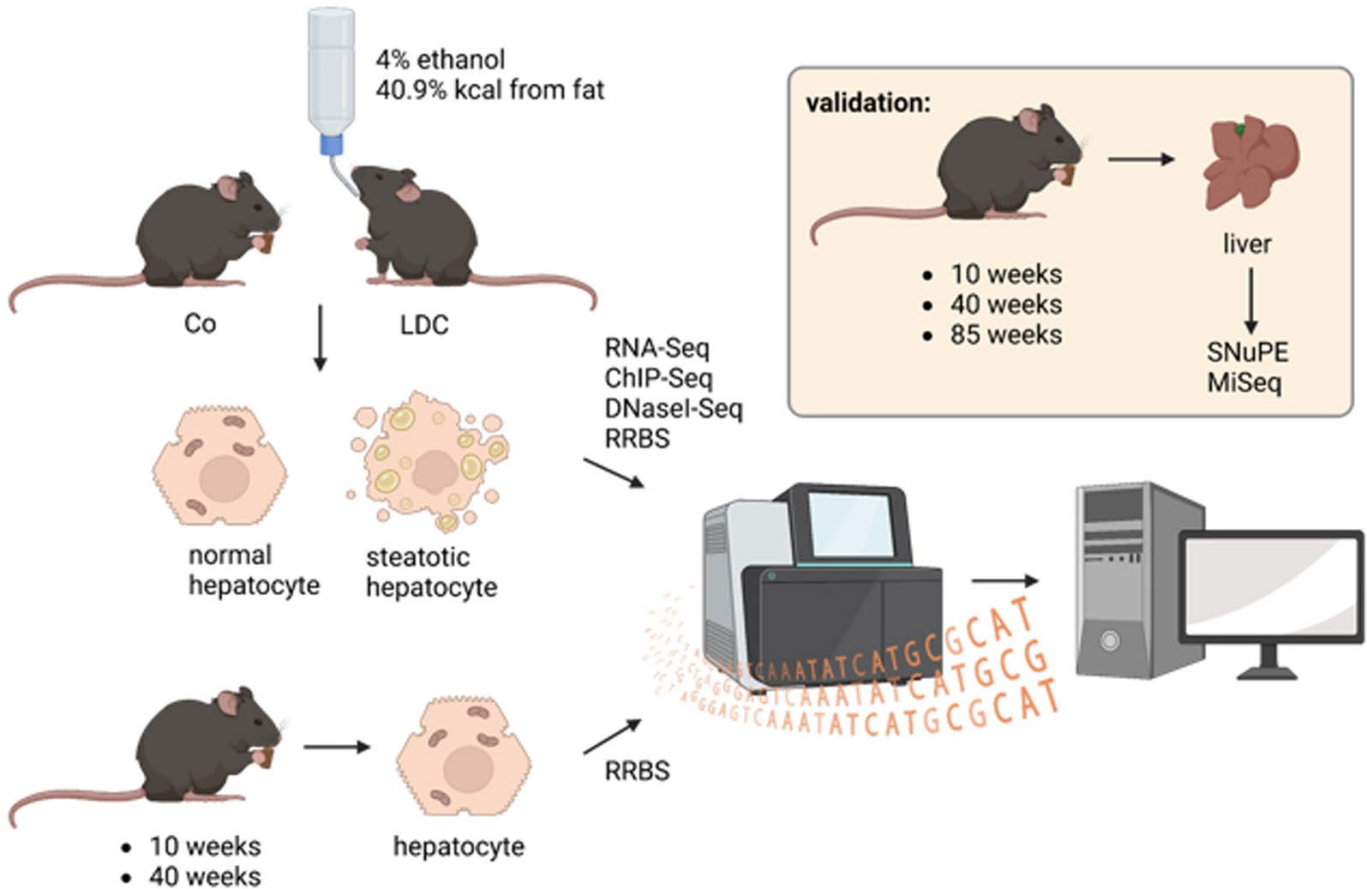
Experimental setup: Upper left: Hepatocytes were isolated from female mice (aged 9 weeks) fed a control diet (Co, 12% kcal from fat) or a Lieber-DeCarli (LDC) diet (4% ethanol and 40.9% kcal from fat) for 6 weeks (n = 2 per group). Thereafter, RNA-seq, ChIP-seq, DNase1-seq, and RRBS were performed for epigenomic analysis. Lower left: Hepatocytes were isolated from young (10 weeks) or mid-aged (40 weeks) mice were fed a control diet and underwent RRBS analyses (n = 4). Top right: Validation was done in liver tissues from young (10 weeks, n = 4), mid-aged (40 weeks, n = 7), and aged (85 weeks, n = 4) female mice fed a control diet by SNuPE and MiSeq. Figure created with *BioRender.com*

We used the Lieber-DeCarli (LDC) diet, containing both high fat and alcohol, model for female mice to investigate transcriptional and epigenomic differences in ex vivo mouse hepatocytes (Fig. 1). After sorting liver hepatocytes from LDC or control (Co) mice we conducted a number of genome-wide assays to profile matched transcriptional and epigenetic activity in these cells.

Body weight change was 209 ± 1.5% in LDC-fed mice compared to 226 ± 11% in control animals. Lipid content of the isolated hepatocytes was 31.33 ± 0.99 lipids/mg liver tissue dry weight in LDC vs. 18.04 ± 0.24 µg in Co analyzed by colorimetric Sulfo-Phospho-Vanillin assay. In the same experiment in which the animals for this manuscript were fed the LDC diet and their hepatocytes were isolated, mice of the same experimental groups were used to obtain bulk tissue and whose livers were examined histopathologically. The latter animals have been investigated previously [15], and it was reported that LDC-fed animals show a microvesicular steatosis linked to an increased steatosis score, although liver weight and AST/ALT levels were not changed. No ballooning of hepatocytes was observed. Hepatocyte death was induced in LDC fed mice, while inflammation was not altered. Further, serum triglyceride and cholesterol levels were increased in the LDC fed animals compared to the control mice.

### Differential gene expression analysis and clustering

We started with the analysis of RNA-seq generated from LDC and Co mice. Differential gene expression analysis of LDC against Co was performed using DESeq2, resulting in 355 differentially expressed genes (DEGs) with adjusted *p*-value < 0.05. Of these, 148 were downregulated and 207 were upregulated (Additional file 2). The 355 DEGs were used to explore possible associations between them. In this direction, a high confidence protein–protein association (PPA) network of the DEGs was constructed using STRING v11 [26] (confidence > 0.7) (Additional file 7: Figs. S1, S2, S3, S4). Thereafter, the DEGs were split into different clusters (k-means clustering with k = 4), so that they could be associated with functional categories. Table 1 shows a subset of enriched functional categories that could be associated with the encoding genes in each of the 4 clusters. All of these four clusters are related to functions, which are altered in MAFLD: mitochondrial dysfunction, extracellular matrix (ECM), alcohol metabolism, and signaling pathways, such as PI3K, JAK-STAT, and MAPK.

**Table 1:** Functional analysis of the DEG clusters

Cluster ID	DEGs per cluster	DEGs overlapping DCDs	DEGs with proximal DMRs	Enriched functions
1	94	1	41	Mitochondrial dysfunction
2	79	0	32	Extracellular matrix collagen
3	61	3	16	Alcohol metabolism
4	84	1	31	Steatohepatitis associated pathways: PI3K-AKT JAK-STAT MAPK

The first column gives the snapshot of the PPA network of each of the 4 clusters built with the DEGs Additional file 7: Fig. S1–4 contain the figures for each cluster, with the cluster ID). The second column gives the number of participating genes (of the DEGs). The third and fourth columns are the overlapping DEGs with differential chromatin domains (DCDs) and differentially methylated regions (DMRs), respectively. Finally, the fifth column provides a summary of the studied functional categories that the genes in the cluster have been linked to

### Analysis of open chromatin and transcription factor binding

**Fig. 2:**
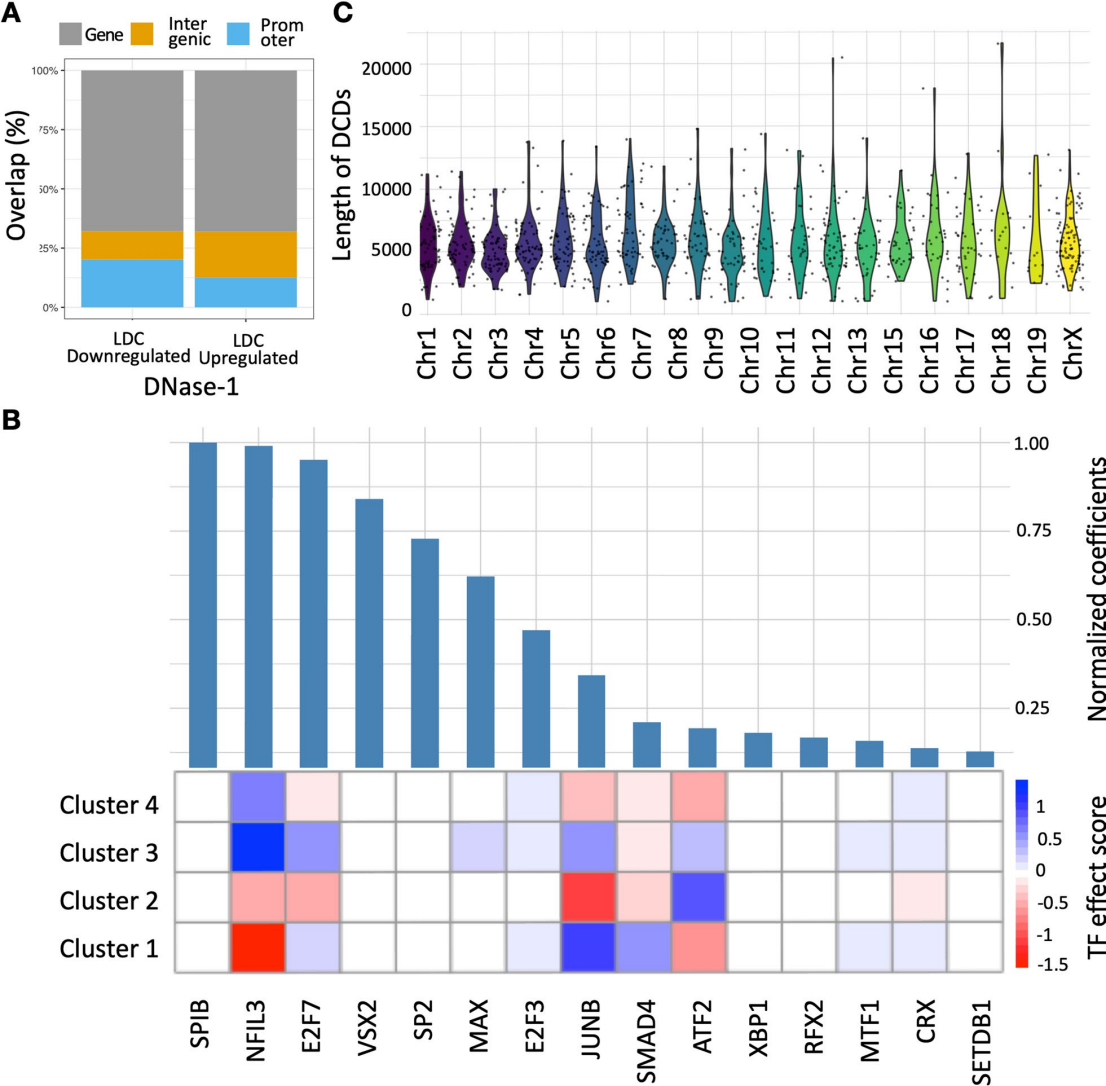
Integrative analysis of epigenomics data reveals involved transcription factors in LDC gene regulation. **a** Distribution of the downregulated and upregulated DNase-1 regions in LDC (over Co) among gene bodies, promoters, and intergenic regions. **b** Integrative analysis of differential DNase1 peaks from deregulated genes lead to the prediction of involved transcription factors using logistic regression. The top panel shows the absolute normalized coefficients of 15 TFs (absolute regression coefficient > 0.125), which are most predictive for the differential genes. The bottom panel shows the TF *effect score* (Eq. 2) of the ranked TFs in each of the 4 clusters. A positive *TF effect score* (blue) signifies stronger regulation of DEGs in the cluster in LDC (over Co) than the *TF effect score* for DEGs in other clusters. **c** Violin plot showing the distribution of the lengths of the Differential Chromatin Domain (DCD) regions over all the chromosomes. The DCDs were obtained using SCIDDO from histone ChIP-seq data (p-value <  = 0.05)

In addition to the analysis of differential expression, DNase1-sequencing was performed for LDC and Co samples. After data processing we obtained regions that show differential DNase-1 abundance between LDC and Co resulting in 58,370 regions with differential accessibility. The latter data was used to investigate the binding of transcription factors (TFs) that may regulate the genes, which show differences in gene expression. Briefly, the peaks were separated into positive or upregulated peak regions (28,358) and negative or downregulated peak regions (30,012) for LDC vs Co based on fold change of the DNase-1 data. Among the downregulated LDC regions, 67.9% overlapped with gene bodies, 20.2% with promoters, and the remaining 11.9% with intergenic regions. The upregulated LDC regions overlapped similarly to 68.05% with gene bodies, 12.6% overlapped with promoters and the remaining 19.4% with intergenic regions (Fig. 2a).

Then, an integrative strategy was conducted using the DYNAMITE method [27] to combine DNase1 and gene expression data. DYNAMITE uses annotation of TF binding motifs in differential epigenome peaks linked to genes to explain differentially expressed genes between LDC and Co via a logistic regression classifier (see *Methods*).

Figure 2b illustrates the top 15 ranked TFs based on the absolute normalized regression coefficients from the DYNAMITE method. We have further investigated whether these 15 TFs show particular strong deviations in their binding behavior for genes according to the 4 gene clusters. The TF effect score (see *Methods*) provides a means of understanding the regulation of the TF in the cluster of interest. A positive effect score therefore means that there is a stronger regulation of cluster genes in LDC with respect to genes in other clusters. Interestingly, NFIL3 showed higher binding preference in LDC samples for genes from *cluster* 1 and *cluster* 2, whereas JUNB showed preferences in LDC for genes in *cluster* 2 and 4. Many of the top TFs did not show differences with respect to the gene clusters, such as *SPIB*, *CSX2*, *SP2*, *XBP1*, and *RFX2* pointing to more general roles of these TFs in the transcriptional response.

### Investigation of larger domains with chromatin changes

The occupancy of six histone modifications (H3K36me3, H3K27ac, H3K27me3, H3K9me3, H3K4me3, H3K4me1) was investigated using ChIP-seq in Co and LDC in order to clarify if there are larger systematic changes in the epigenome of steatotic samples. After chromatin state annotation with ChromHMM, the SCIDDO method was used to predict differential chromatin domains (DCDs), which are larger genomic regions that show a consistent difference in chromatin states between the epigenome (Additional file 3). Overall, SCIDDO reported 1018 DCDs, which were overlapping 272 genes. Among these 272, 8 were also differentially expressed in the RNA-seq data, when considering the closest gene within a window of 50 KB.

Functional categorization (using gProfiler [20]) of these 272 genes shows enrichment of molecular functions, such as *intracellular calcium activated chloride channel activity* ([39], and reviewed in [40]). *Cell adhesion* and *developmental processes* were enriched as biological processes. *Col4a2*, among the 8 DEGs, (also intersecting with DMRs) was reported to be significantly correlated with hepatocarcinogenesis, HCC progression, and prognosis [41].

The distribution of the lengths of the DCD regions for each chromosome is shown in**. **Figure 2c. Overall, only a few larger genomic domains showed significant changes in histone modifications between LDC and Co, epigenomic changes were mostly limited to smaller regions.

### Differential methylation in young, aged, and steatotic livers

**Fig. 3:**
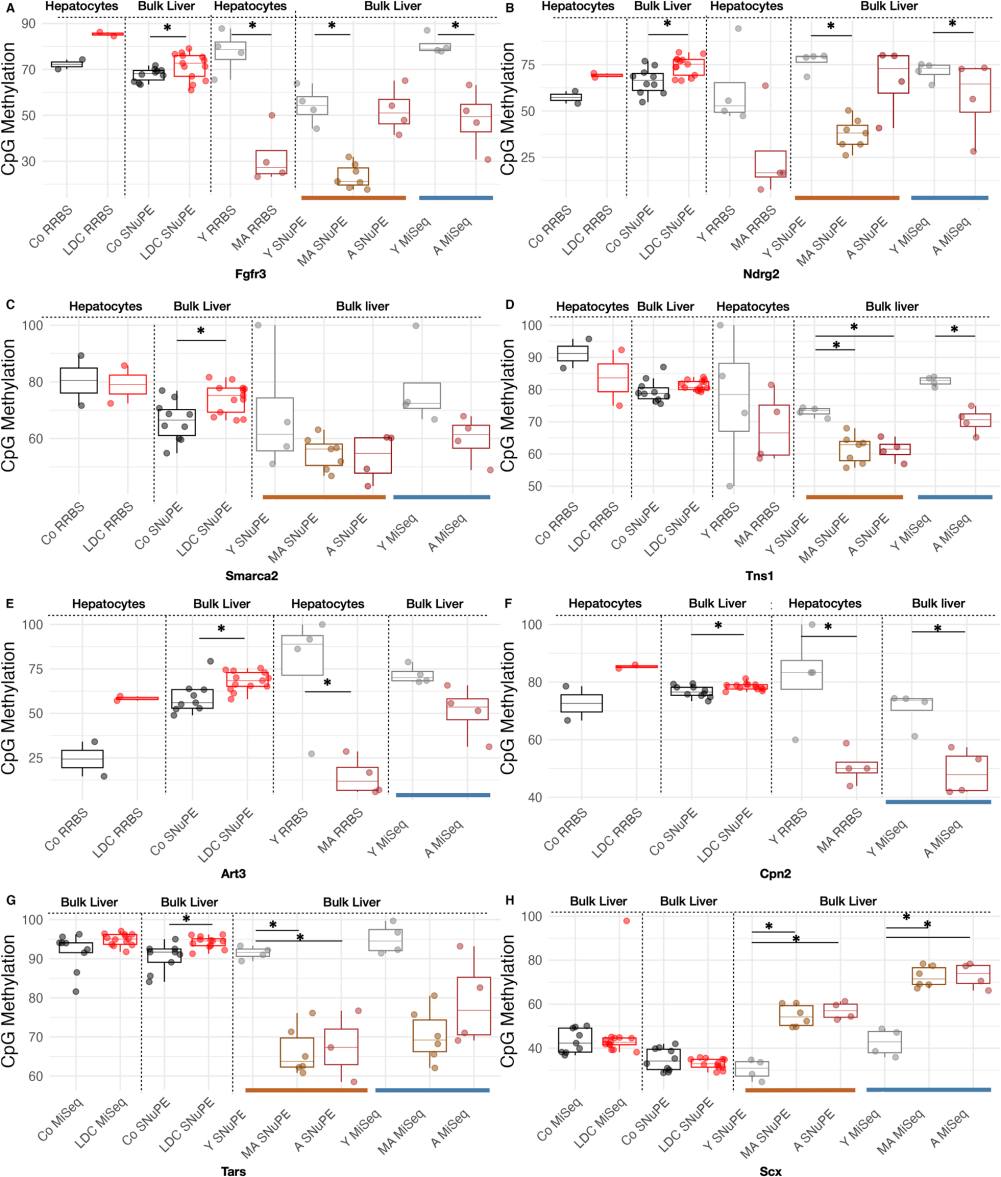
Analysis of CpG methylation in established and novel aging associated loci. **A**–**F** Analysis of CpG methylation status of selected Stubbs Loci (see Table 2) in LDC (over Co) mice from liver hepatocytes (using RRBS) and in bulk liver (using SNuPE). The CpG methylation is compared to bulk liver from aging mice using SNuPE and MiSeq. **G**, **H** The CpG methylation of the new aging loci was also measured in LDC (over Co) mice and compared to aging bulk liver using SNuPE and MiSeq. *t test* (parametric, unpaired) was used to compare and evaluate the significance between groups Co vs LDC and young (Y = 10 wks), mid-aged (MA = 40 wks) vs aged (A = 85 wks) for each of the technologies, in female mice. All differences that are significant (* *p* < 0.05) are marked in the plot. In each case, comparisons were made only between two groups (LDC vs. Co, Y vs. A or Y vs. MA for each technology). The gene associated with the measured CpG is mentioned at the bottom of each plot. The source of the sample and their age are mentioned on top. For better comparison, methylation index values obtained from SNuPE analyses were normalized to the range of [0,100]. RRBS data was excluded when read coverage for the respective CpG was below 3 (concerns *Smarca2, Inpp5a*). SNuPE analysis of CpGs with low read coverage were excluded

Human steatotic livers share alterations in function, cellular signaling, as well as in metabolism with aged livers [42]. It has been described that on the one hand liver disease reveals specific alterations in DNA methylation and on the other hand epigenetic aging signatures are affected by dietary conditions. Thus, the impact of LDC feeding on epigenetic aging marks was investigated. According to the human epigenetic clock described by Horvath [13], Stubbs et al. [14] reported an RRBS-based murine epigenetic clock [14], which allows to investigate more precisely aging effects in mouse models. Based on their work, liver-specific CpG loci obtained from mid-aged mice (41 weeks old) were selected based on an absolute methylation difference of more than 30% (FDR-adjusted p-value < 0.1, Additional file 4) linked to the genes *Tns1*, *Fgfr3*, *Art3*, *Cpn2*, and *Inpp5a* (Fig. 3, Additional file 7: Fig S5, Table 2). In order to assess whether these altered methylations found in liver tissues can be observed in isolated hepatocytes or whether they are rather a result from an altered cell composition with age, we performed RRBS analyses of hepatocytes isolated from young (10 weeks) and mid-aged (40 weeks) mice. RRBS data confirmed a lower degree of methylation in hepatocytes from mid-aged compared to young animals for *Fgfr3*, *Ndrg2*, *Tns1*, *Art*, and *Cpn2*. RRBS data were excluded when the number of reads (coverage) for the respective CpG was below 3, as it was the case for *Smarca2* (Fig. 3c) and *Inpp5a* (Additional file 7: Fig. S5d).

**Table 2:** Investigated CpGs with association to genetic age

Position	Gene	Annotation	Source
chr 4: 141,251,015–141,251,016	*Arhgef19*	(+) exon 13	New locus
chr 15: 11,383,946–11,383,947	*Tars*	(+) 3’UTR	New locus
chr 9: 85,324,737–85,324,738	*Tent5a*	(+) exon 3	New locus
chr 15: 76,457,712–76,457,713	*Scx*	( +) exon 1	New locus
chr 6: 94,667,253–94,667,254	*Lrig1*	(+) intron	Stubbs et al., 2017 [14]
chr 5: 33,729,707–33,729,708	*Fgfr3*	(+) exon 5	Stubbs et al., 2017 [14]
chr 16: 30,260,867–30,260,868	*Cpn2*	(−) exon 2	Stubbs et al., 2017 [14]
chr 1: 73,959,153–73,959,154	*Tns1*	(−) intron	Stubbs et al., 2017 [14]
chr 14: 51,908,372–51,908,373	*Ndrg2*	(−) exon 9	Stubbs et al., 2017 [14]
chr 19: 26,640,521–26,640,522	*Smarca2*	(+) intron	Stubbs et al., 2017 [14]
chr 5: 92,364,789–92,364,790	*Art3*	(+) intron	Stubbs et al., 2017 [14]
chr 4: 154,557,099–154,557,100	*Prdm16*	(−) exon/intron	Stubbs et al., 2017 [14]
chr 7: 139,559,949–139,559,950	*Inpp5a*	(+) intron	Stubbs et al., 2017 [14]

The first, second, and third columns give the position and the associated annotation of the respective CpG. The fourth column denotes if a CpG was discovered as a new locus or reported before [14]. mm10 was used as the reference genome

Further validation was done for these CpGs as well as for *Smarca2* and *Inpp5a* via MiSeq and SNuPE analyses of liver tissues from young, mid-aged, and aged (85–86 weeks) mice. These analyses confirmed a lower degree of methylation as reported by Stubbs et al. [14] in liver tissue not only from mid-aged, but also from aged mice: CpGs linked to *Fgfr3*, *Ndrg2*, *Tns1, Art3, and Cpn2* showed significant hypermethylation in livers from young compared to (mid-)aged mice (see Fig. 3A, B, D–F respectively), while values for *Smarca2* did not reach statistical significance (Fig. 3c).

Subsequently, we performed RRBS and identified new CpG loci (Table 2) in hepatocytes with a consistently altered methylation in aged livers: two hypomethylated CpGs linked to *Arhgef19* and *Tars* (Additional file 7: Fig. S5A, Fig. 3G), and one hypermethylated CpG linked to *Tent5a* and *Scx*, respectively (Additional file 7: Fig. S5B, Fig. 3H), (also Additional file 5).

Comparing aging-related CpG methylation with CpG methylation in hepatocytes or livers from LDC-fed animals, none of the sites showed an aging-related pattern: *Scx* showed a significant CpG hypermethylation in aged (both SNuPe and MiSeq) over young (Fig. 3H), while it was not significantly different in LDC (over Co). *Tars* (Fig. 3G), *Fgfr3* (Fig. 3A), *Ndrg2* (Fig. 3B), *Art3* (Fig. 3E), and *Cpn2* (Fig. 3F) showed a lower CpG methylation in livers from aged than young mice, but the opposite in livers from LDC *vs*. control-fed animals. *Tns1* (Fig. 3D) CpG methylation was reduced in aged livers, but no clear effect could be observed upon the LDC diet. The *Arhgef19* (Additional file 7: Fig. S5A) locus had significantly higher CpG methylation in young (over aged), as detected by SNuPE experiments, with similar tendency in MiSeq experiments.

A CpG of the *Prdm16* gene was used as a consistently highly methylated control (Additional file 7: Fig. S6). Though *Tent5a* (Additional file 7: Fig S5b) did not have significant differences in aging or LDC, the aged samples in tendency showed a higher extent of methylation (compared to young).

## Discussion

In this study, different layers of the epigenome were analyzed in hepatocytes isolated from mice that were fed a diet containing alcohol and high fat. One limitation of the study is that the epigenome assays were produced from two mouse replicates *(n* = *2 per group)*. To the best of our knowledge, no comparable data have been reported in the literature. In order to decipher which epigenetic mechanisms cause the observed transcriptional alterations, we investigated expression, DNA methylation, histone modifications, and chromatin changes. We acknowledge that variation in chromatin accessibility or DNA methylation does not imply the changes observed in gene expression. However, keeping these assumptions in mind and with the help of integrative approaches, biological interactions of different epigenetic and transcriptional mechanisms could be identified for LDC and might be also relevant in human disease.

The four clusters resulting from differential gene expression analysis are all relevant for the pathogenesis of steatosis and steatohepatitis (Table 1). *Cluster* 1 depicts mitochondrial dysfunction, although being based on only a few DEGs. In general, NAFLD and AFLD are associated with mitochondrial dysfunction [43, 44]. NAFLD is associated with an increased mtDNA mutation rate and mtDNA variability drives NAFLD progression [45], including the genes differentially expressed in LDC in the current study. *Cluster* 2 represents changes in genes associated with ECM/collagen. Alterations in the ECM of steatotic livers are well known to contribute to morphological changes in the liver in ALD as well as NAFLD. Fibrosis is characterized by collagen deposition as well as ECM remodeling. *Cluster* 3, which is associated with metabolism, e.g. alcohol metabolism, well reflects the LDC model used in this study. *Cluster* 4 includes genes of several PI3K-AKT, JAK-STAT, and MAPK pathways by a KEGG analysis. All of these pathways can be connected with steatohepatitis, although most of the genes in this cluster have not been quite studied with respect to the liver. By using a machine learning approach that uses TF binding profiles and the differential gene expression profiles from LDC and Co [27], we were able to predict specific transcription factors which might cause the observed DEG profile for specific clusters, respectively. Based on the TF prediction we found a number of TFs that have been previously linked to steatosis. *NFIL3* and *JUNB* have been reported to play a role in all stages of human NAFLD [46]. *NFIL3* was described as part of an enhancer hotspot associated gene in NASH-prone livers [47] and mechanistically affects gluconeogenesis in hepatocytes [48, p. 3] and lipid accumulation [49]. The preference of *JunB* for ECM cluster genes (*cluster* 2) confirmed a recently described positive correlation of *JunB* levels with liver fibrosis in human and murine samples in hepatic stellate cells [50]. Regarding hepatocytes, a recent study found *JUN*, *JUND*, and *JUNB* as the three top predicted transcription factors in NASH [51]. The paper also suggested a major crosstalk between hepatocytes and non-parenchymal cells during NASH progression. This crosstalk comprises genes coding, e.g., for cell adhesion molecules (integrins) or phosphatases as found in *cluster* 2. The most strongly upregulated gene in this cluster is *Krt23*, which has been described to be the gene that has the clearest upregulation in livers from alcoholic hepatitis patients compared to NASH patients [3]. Genes encoding ECM-regulating proteins, such as *Timp3*, which have been shown to be protective in steatosis and HCC when overexpressed in hepatocytes, were downregulated in our study [52].

*E2F7* has been previously found to inhibit liver tumor growth and through regulation of polyploidy also be involved in different hepatic diseases. *ATF2* has been previously linked to non-alcoholic fatty liver disease [53]. *SPIB* has not been linked to liver pathogenesis so far, but belongs to the group of interferon regulatory factors and therefore is involved in inflammatory conditions [54]. Taken together, the TF enrichment analysis well recapitulates what is known from the literature regarding human and murine fatty liver disease.

Since the characteristics of a NAFLD liver were suggested to recapitulate the main aspects of the aged liver phenotype, previously published DNA methylation aging marks were examined in LDC hepatocytes. Although epigenetic clocks are a relatively novel tool, they are already established as highly valuable age prediction methods [55]. Several studies identified altered DNA methylation CpG sites in liver tissues from patients with obesity of type 2 diabetes, both conditions being associated with MAFLD [56–59]. Vice versa, epigenetic aging signatures are slowed by caloric restriction in mice [60]. However, interestingly, Horvath et al. [13] observed a strong correlation between high body mass index and the epigenetic age of liver tissue, but no association with the NAFLD activity score (NAS), steatosis, inflammation, or fibrosis [13], which might explain the lack of DNA methylation age marks in the LDC model in the current study. In general, the relationship between aging and fatty liver disease is controversial [61, 62]. According to the inflamm-aging theory, aging promotes liver inflammation [63]. However, although aging has been reported to increase hepatic lipid accumulation [17, 64–66], aging per se has no effect on steatosis in mid-aged mice [67]. Interestingly, there are inconclusive data on the progression of steatosis to NASH in the very elderly [68]. The discrepancy between the studies was suggested to be due to the fact that some of the studies were conducted in moderately aged rather than in very old mice and humans [62].

Our data confirmed reduced methylation for CpG loci as originally measured by Stubbs et al. [14] both for liver tissue as well as for isolated hepatocytes from mid-aged mice and could also confirm them for liver tissue from aged animals. Interestingly, our data do not suggest similarities in the methylation status of respective loci between aging and steatosis. In contrast, methylation patterns of livers or hepatocytes from LDC-fed animals rather had similarities with those from young animals. This is also true for newly identified loci characteristic for aged hepatocytes and livers.

Taken together, this study highlights four clusters of differentially expressed genes being relevant in the LDC model. The regulation of transcription of these genes was shown to be affected by chromatin changes and transcription factors. Steatosis was not associated with DNAm age in this model.

## Supplementary Information

**Additional file 1.** Primers used for bisulfite-based PCR with subsequent SNuPE and local deep bisulfite sequencing (MiSeq). For MiSeq universal TruSeq adaptor sequences were attached to the specific sequences *in silico* and used as fusion primers in the PCR.

**Additional file 2.** The differentially expressed genes in LDC (over Co).

**Additional file 3.** BED file with the differential chromatin domains in LDC (compared over Co).

**Additional file 4.** Differentially Methylated Regions (with FDR < 0.05) in LDC.

**Additional file 5.** CpG methylation for all the sites from various technologies.

**Additional file 6.** Excel file with Accession IDs of all sequence data used in the study.

**Additional file 7.** Additional figures.

## Data Availability

All code and the supplementary data to make the figures in the manuscript are available from https://github.com/SchulzLab/Liver-Steatosis-Supplementary. Additional file 6 provides the accession IDs to all sequencing data used in the study.
