# Supplementary material for: Alterations in the hepatocyte epigenetic landscape in steatosis
Source: Epigenetics Chromatin. 2023 Jul 6;16:30. doi: 10.1186/s13072-023-00504-8 (PMC10324225; doi:10.1186/s13072-023-00504-8)
Supplement: Supplementary file 7 — Additional file 7. Additional figures. [file 13072_2023_504_MOESM7_ESM.pdf]

Additional Figures: Alterations in the hepatocyte epigenetic landscape in steatosis

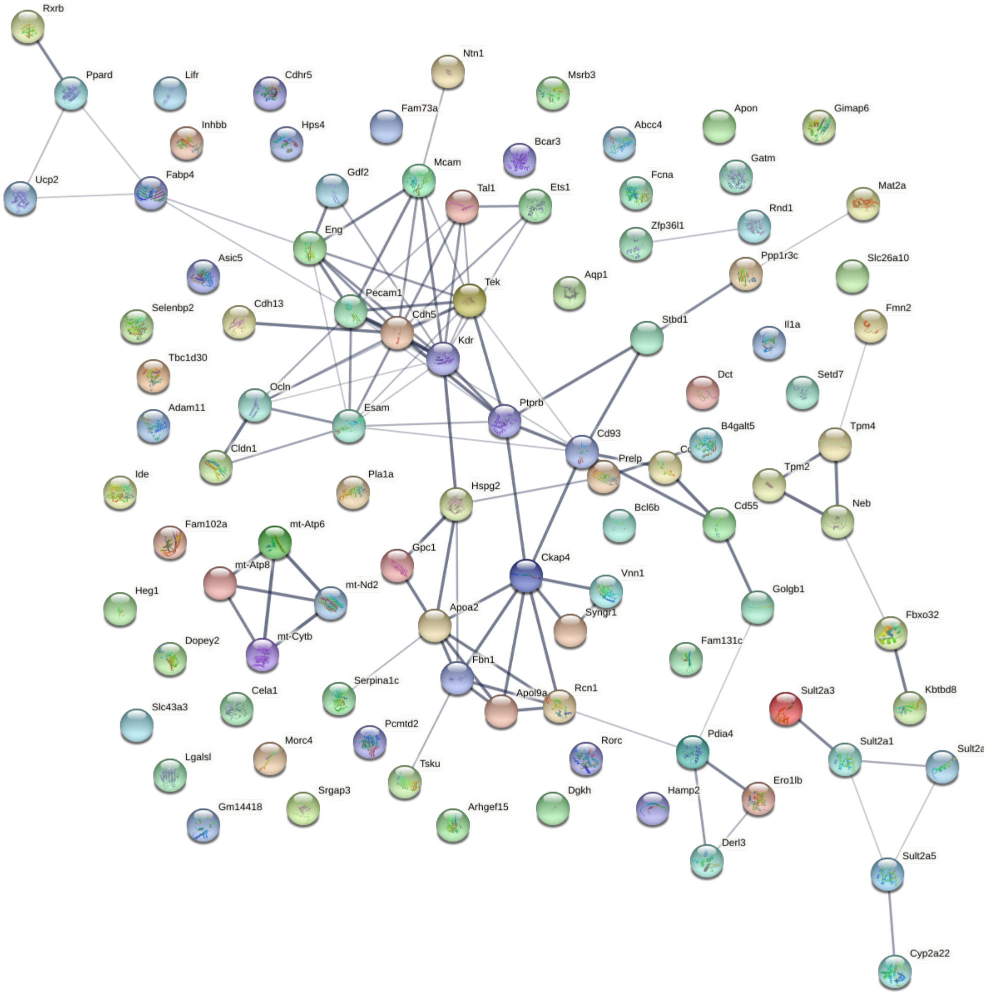

Additional Figure S1: The 1st cluster of the PPI network. The constituent genes in this cluster are enriched in Mitochondrial dysfunction.

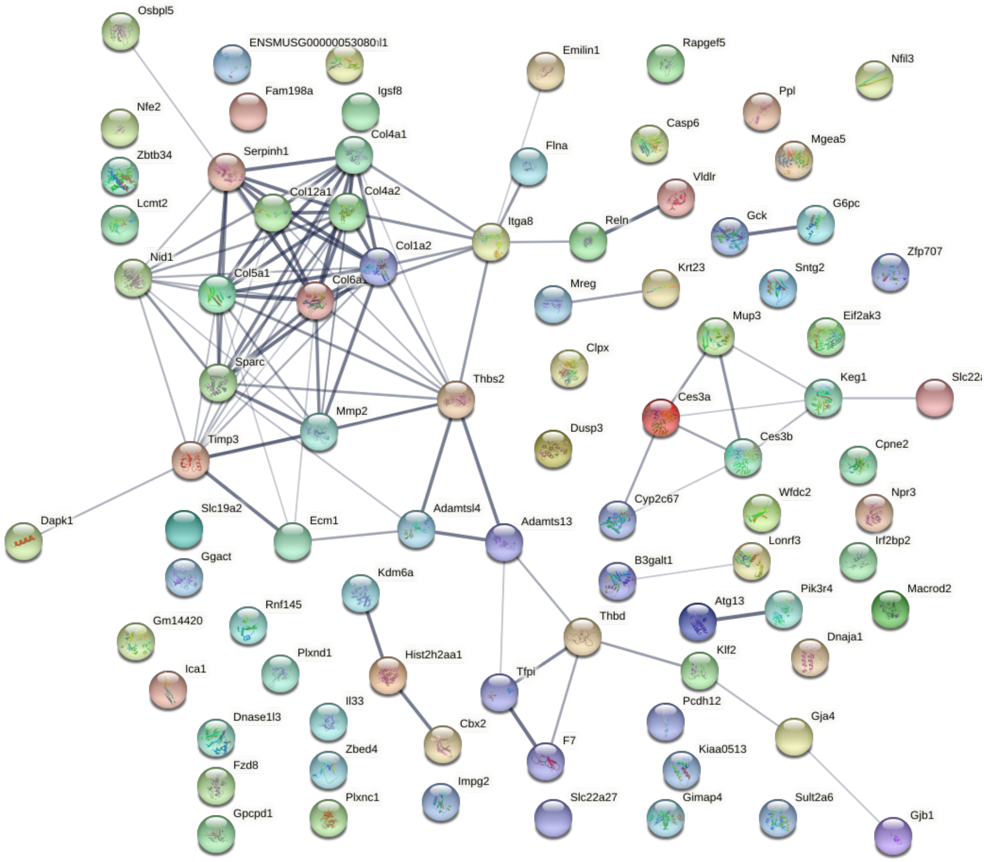

Additional Figure S2: The 2nd cluster of the PPI network. The constituent genes are enriched in extra cellular matrix collagen.

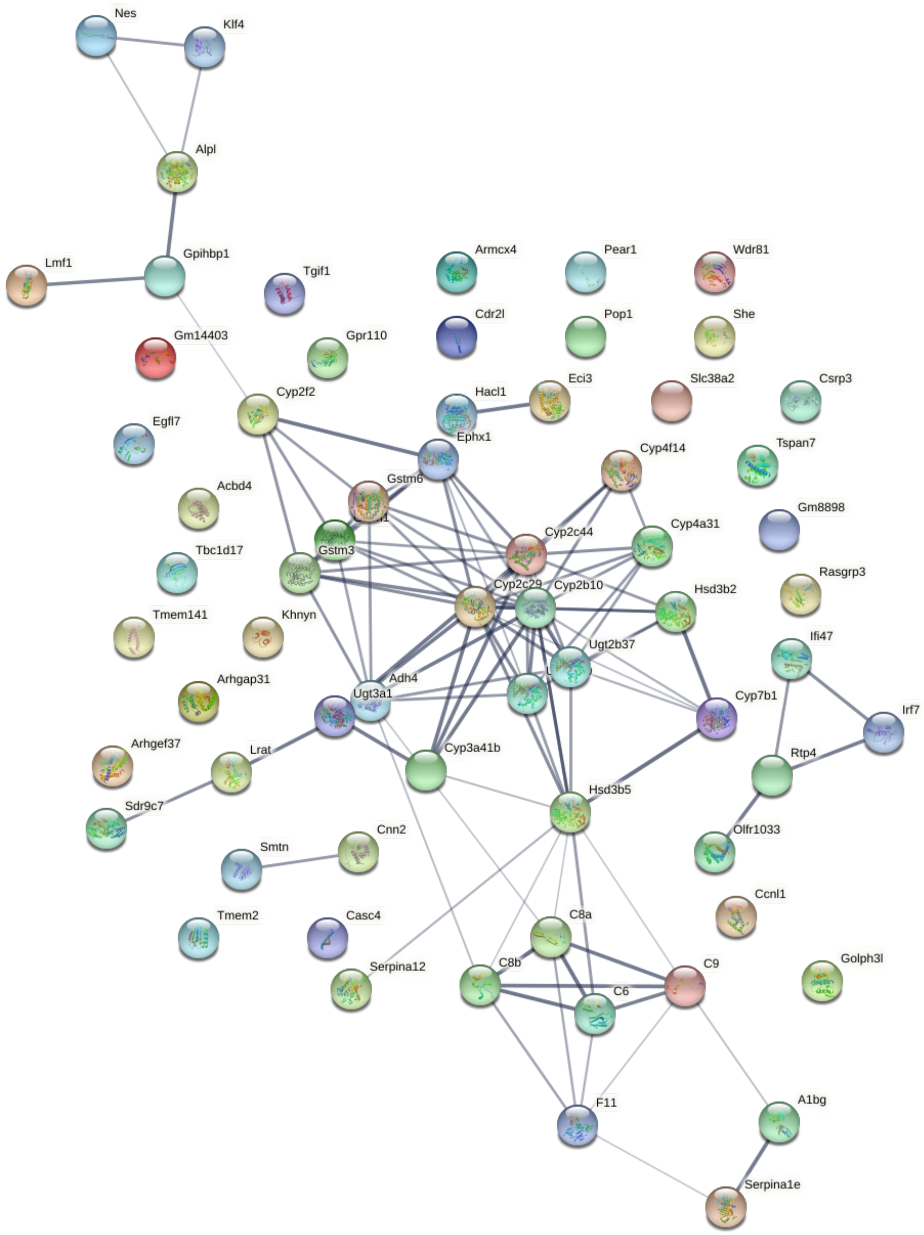

Additional Figure S3: The 3rd cluster of the PPI network. The constituent genes are enriched in alcohol metabolism.

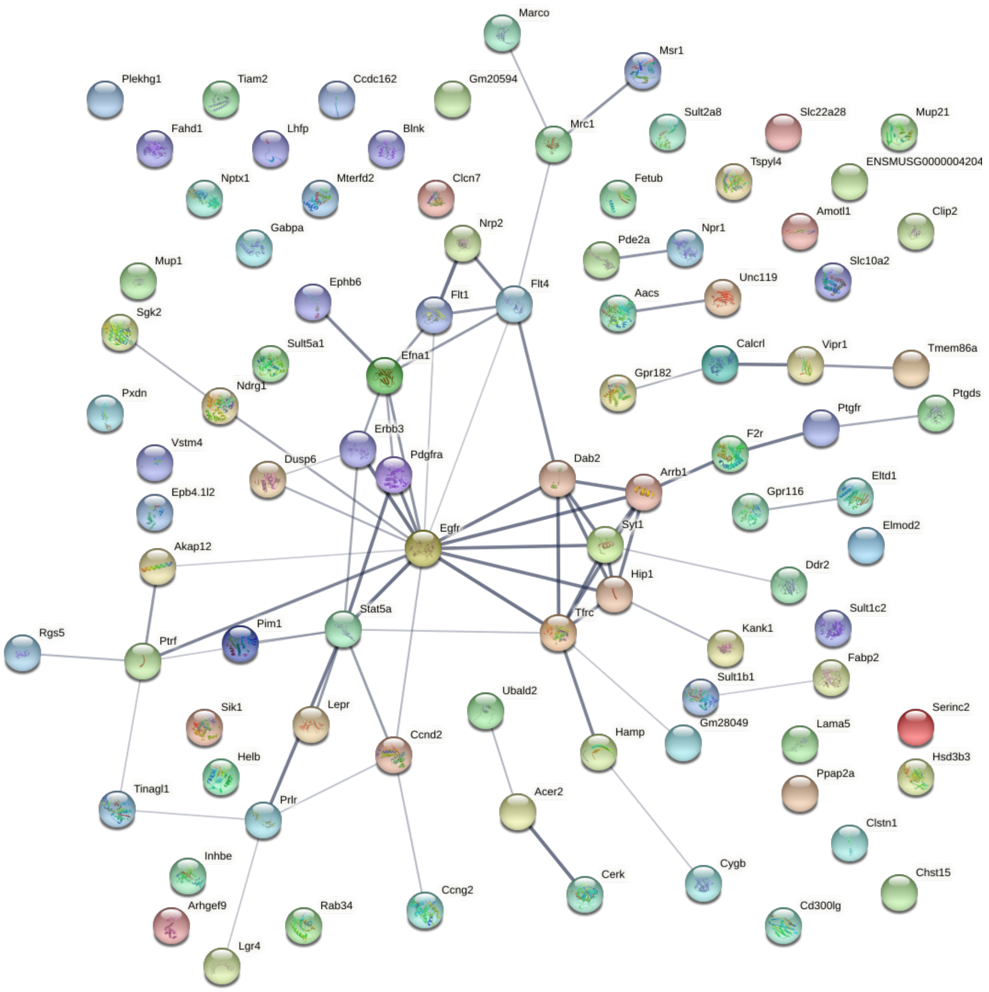

Additional Figure S4: The 4th cluster of the PPI network. The constituent genes are enriched in steato-hepatitis associated pathways.

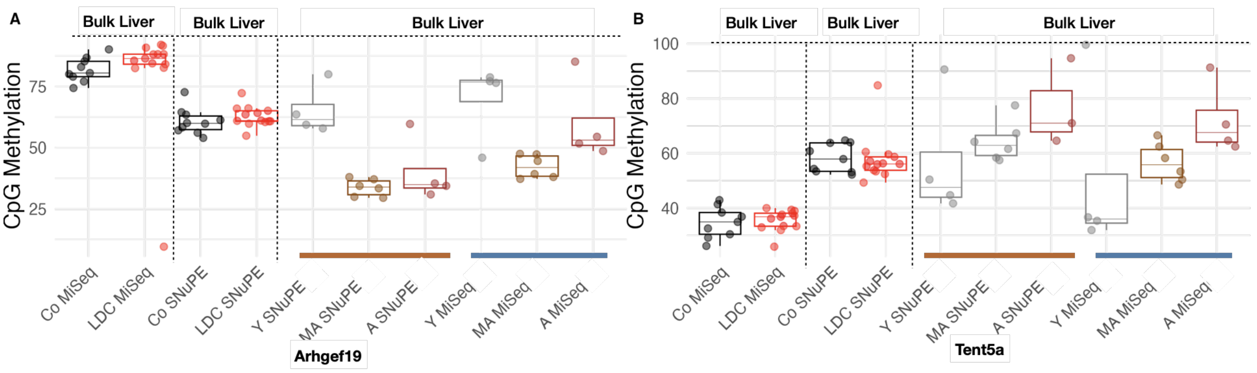

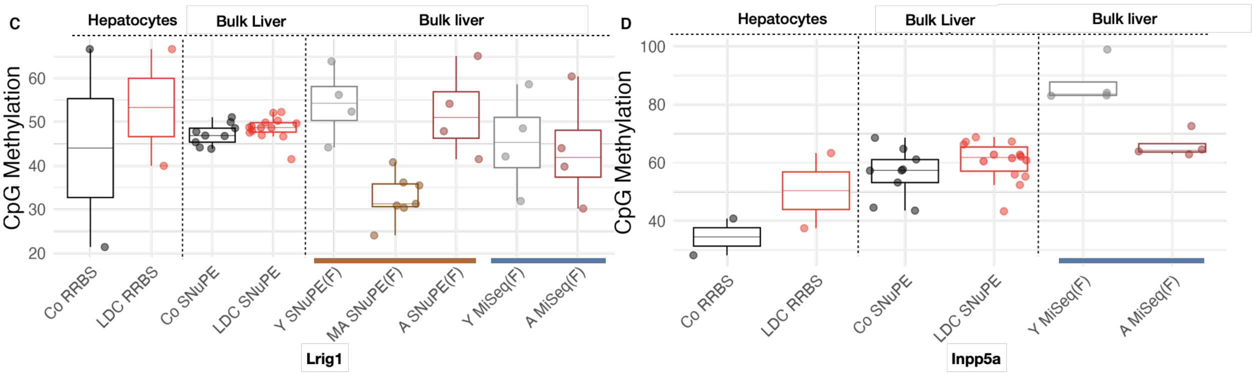

Additional Figure S5: The CpG methylation status of the Stubbs Loci (as shown in Table 1) in LDC (over Co) mice from liver hepatocytes (using RRBS) and in bulk liver (using SNuPE). The CpG methylation is compared to bulk liver from aging mice using SNuPE and MiSeq (I, J). The CpG methylation of the new aging loci was also measured in LDC (over Co) mice and compared to aging bulk liver using SNuPE and MiSeq. (K, L). t test (parametric, unpaired) was used to compare and evaluate the significance between groups Co vs LDC and young (Y) , mid-aged (MA) vs aged (A) for each of the technologies, in both (M) male and (F) female. The gene associated with the measured CpG is mentioned at the bottom of each plot. The source of the sample and the age are mentioned on top. All differences that are significant (* $p < 0.05$) are marked in the plot. In each case, comparisons were made between two groups (LDC vs. Co, Y vs. A or Y vs. MA for each technology). For better comparison, methylation values obtained from SNuPE analyses were plotted as methylation index multiplied by 100. RRBS data was excluded when read coverage for the respective CpG was below 3 (concerns Smarca2). SNuPE analysis with low coverage is excluded from the figure as well.

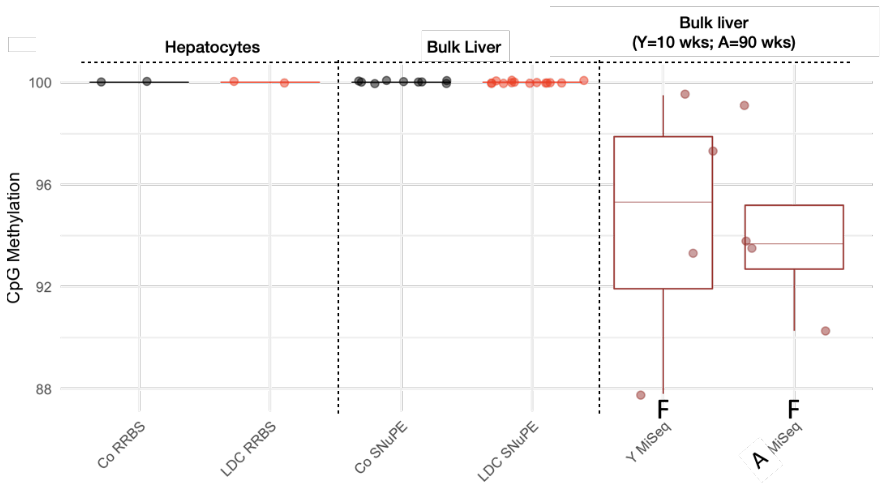

Additional Figure S6: Validation of age associated CpG loci: Prdm16 with SNUPE and MiSeq and comparison of CpGs in aging (from miseq and RRBS). The differences of the groups are non significant in these loci. t test (parametric, unpaired) was used to compare and evaluate the significance between groups. * $p <= 0.05$.
